# (-)-Epigallocatechin-3-gallate suppresses hepatic preneoplastic lesions developed in a novel rat model of non-alcoholic steatohepatitis

**DOI:** 10.1186/2193-1801-2-690

**Published:** 2013-12-27

**Authors:** Takafumi Sumi, Yohei Shirakami, Masahito Shimizu, Takahiro Kochi, Tomohiko Ohno, Masaya Kubota, Makoto Shiraki, Hisashi Tsurumi, Takuji Tanaka, Hisataka Moriwaki

**Affiliations:** Department of Internal Medicine, Gifu University Graduate School of Medicine, Gifu, Japan; Department of Tumor Pathology, Gifu University Graduate School of Medicine, Gifu, Japan; Department of Internal Medicine/Gastroenterology, Gifu University Graduate School of Medicine, 1-1 Yanagido, Gifu, 501-1194 Japan

**Keywords:** Non-alcoholic steatohepatitis, Liver tumorigenesis, Oxidative stress, Inflammation, EGCG

## Abstract

**Purpose:**

Non-alcoholic fatty liver disease (NAFLD) ranges from simple steatosis to non-alcoholic steatohepatitis (NASH). NASH, which is accompanied by increased oxidative stress and inflammation in the liver, is associated with hepatic carcinogenesis. Green tea catechins (GTCs) possess anti-oxidant, anti-inflammatory, and cancer-preventive properties. In this study, we investigated whether (-)-epigallocatechin-3-gallate (EGCG), a major component of GTCs, inhibits NAFLD/NASH-related liver tumorigenesis.

**Methods:**

Male 8-week-old Sprague–Dawley (SD) rats were administered a single intraperitoneal injection of a hepatic carcinogen diethylnitrosamine (DEN, 30 mg/kg body weight) and then fed a high-fat diet (HFD) for 7 weeks. The rats were also provided tap water containing 0.01% or 0.1% EGCG during the experiment.

**Results:**

At sacrifice, the livers of SD rats treated with DEN and HFD exhibited marked development of glutathione *S*-transferase placental form (GST-P)-positive foci, a hepatic preneoplastic lesion, and this was associated with hepatic steatosis, oxidative stress and inflammation, and hepatocyte proliferation. EGCG administration, however, inhibited the development of GST-P-positive foci by decreasing hepatic triglyceride content, reducing hepatic fibrosis, lowering oxidative stress, attenuating inflammation, and inhibiting excessive hepatocyte proliferation in DEN- and HFD-treated SD rats. These findings suggest that the experimental model of SD rats treated with HFD and DEN, in which histopathological and pathophysiological characteristics of NASH and the development of hepatic premalignant lesions were observed, might facilitate the evaluation of liver tumorigenesis associated with NAFLD/NASH.

**Conclusions:**

Administering EGCG, a GTC, might serve as an effective chemoprevention modality for NAFLD/NASH-related liver tumorigenesis.

## Background

Non-alcoholic fatty liver disease (NAFLD), which is considered a hepatic manifestation of the metabolic syndrome, is currently one of the most common chronic liver diseases worldwide. NAFLD is classified into simple steatosis and non-alcoholic steatohepatitis (NASH), the latter being a severe condition of inflamed fatty liver that can, in time, lead to hepatic fibrosis, cirrhosis, hepatocellular carcinoma (HCC) development, and result in increased mortality (Chiang et al., 2011; Cusi, 2012; Siegel and Zhu, 2009). Steatohepatitis has been indicated by epidemiological studies to be a significant risk factor for developing HCC, at an annual HCC incidence of 2%–3% in patients with NASH (Adams et al., 2005; Ascha et al., 2010). In 1998, Day and James proposed a “two-hit theory” to explain NAFLD/NASH pathogenesis: the first hit, hepatic steatosis, increases the sensitivity of the liver to proinflammatory insults, while the second hit involves oxidative stress (Day and James, 1998). Oxidative stress, which is associated with HCC development (Suzuki et al., 2013), is substantially higher in NASH patients than in NAFLD patients and normal control subjects (Sanyal et al., 2001). Moreover, tumor necrosis factor (TNF)-α and interleukin (IL)-6, both of which are major proinflammatory cytokines, play a critical role in obesity-related steatohepatitis and subsequent liver tumorigenesis (Park et al., 2010).

Several animal models that mimic the pathophysiological mechanisms associated with NAFLD/NASH-related liver carcinogenesis have been developed recently (Hebbard and George, 2011; Schattenberg and Galle, 2010; Kochi et al., 2013). For instance, *db/db* mice, which exhibit obesity, diabetes, dyslipidemia, and severe steatosis, are susceptible to liver tumorigenesis induced by a hepatic carcinogen diethylnitrosamine (DEN), and thus are regarded as useful animal models for investigating pathobiology of NAFLD/NASH-related liver carcinogenesis and for screening the chemopreventive effects of various compounds either synthetic or natural on NAFLD/NASH-related liver carcinogenesis (Iwasa et al., 2010; Shimizu et al. 2011a,2011b,2011c). Recently, Wang et al. (Wang et al. 2009) reported that NASH induced by a high-fat diet (HFD) promoted DEN-initiated early hepatocarcinogenesis in Sprague–Dawley (SD) rats and that this was associated with increased oxidative stress and inflammation. This animal model is also useful for investigating the efficacy of certain types of synthetic compounds and/or phytochemicals in preventing NASH-promoted liver carcinogenesis (Wang et al., 2010).

The prevalence of NAFLD/NASH has risen recently in parallel with the dramatic increase in obesity and its related metabolic abnormalities, especially diabetes mellitus (Chiang et al., 2011; Cusi, 2012; Siegel and Zhu, 2009), indicating an urgent requirement for developing an effective strategy to treat NAFLD/NASH and, consequently, to prevent NAFLD/NASH-related liver carcinogenesis. A recent randomized trial (Sanyal et al., 2010) has shown that treatment with vitamin E, an antioxidant, reduces steatosis and lobular inflammation in the liver of NASH patients. Reducing oxidative stress and inhibiting the inflammation induced by obesity and steatosis are also effective in preventing obesity- and diabetes-related hepatotumorigenesis (Shimizu et al. 2013,2012). These reports suggest that targeting oxidative stress and chronic inflammation is an optimal strategy for preventing NAFLD/NASH-related liver carcinogenesis.

Green tea catechins (GTCs) might be one of the most promising candidate compounds for preventing NAFLD/NASH-related liver carcinogenesis because they are considered to protect against metabolic disorders such as NAFLD (Masterjohn and Bruno, 2012; Thielecke and Boschmann, 2009) and also display cancer chemopreventive properties in various tissues, including the liver (Shimizu et al. 2011b; Kochi et al., 2013; Yang et al., 2009). In this study, we developed a novel rat model of NAFLD/NASH-related carcinogenesis and investigated the potential capacity of (-)-epigallocatechin-3-gallate (EGCG), a major component of GTCs, to inhibit the occurrence of HFD- and DEN-induced glutathione *S*-transferase placental form (GST-P)-positive foci, an indicator of preneoplastic HCC lesions in rats (Tsuda et al., 2003; Ando et al., 2007).

## Results

### General observations

At the end of the experimental period, the rats in the 3 groups exhibited no significant differences with respect to the mean body, liver, and kidney weights (Table 1). Histopathological examination revealed that administering EGCG produced no detectable toxic effects on critical organs including the liver, kidney, and spleen (data not shown).

**Table 1:** **Body and organ weights of the experimental rats**

Group No.	EGCG	No. of rats	Body weight (g)	Relative organ weight (g/100 g body weight)	
				Liver	Kidneys
1	-	7	512.7 ± 37.8^a^	3.8 ± 0.5	0.6 ± 0.4
2	0.01%	6	495.5 ± 29.0	3.9 ± 0.6	0.7 ± 0.3
3	0.1%	6	501.6 ± 44.3	3.5 ± 0.2	0.6 ± 0.3

^a^Mean ± SD.

### Effects of EGCG on hepatic steatosis and serum ALT levels in rats

**Figure 1:**
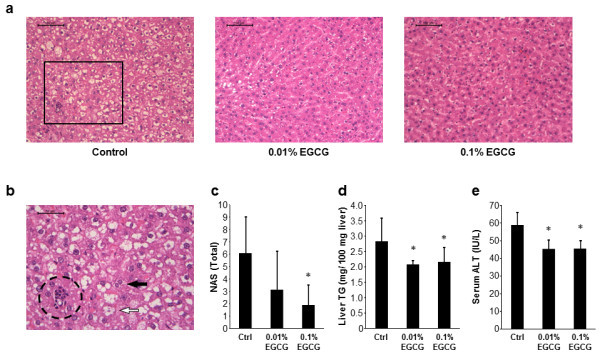
**Hepatic histopathology, intrahepatic levels of triglycerides, and serum levels of ALT. (a)** and **(b)** H&E staining of liver sections from the experimental rats. **(a)** Representative photomicrographs of liver sections of rats treated DEN/HFD with or without EGCG. Bars, 100 μm. **(b)** An enlarged photo of the liver section from a rat treated DEN/HFD without EGCG (an enclosed area with square in Figure 1a). Note ballooned hepatocytes (indicated by the white arrow), Mallory-Denk bodies (indicated by the black arrow), and focus of inflammatory cells (circled by dotted line). Bars, 50 μm. **(c)** The NAS was determined from histopathological analysis. **(d)** Hepatic lipids were extracted from frozen liver samples of rats in the 3 groups, and the triglyceride (TG) levels were measured. **(e)** Blood samples were collected at sacrifice and the serum levels of alanine aminotransferase (ALT) were determined. The values are expressed as mean ± SD. *P < 0.05. Ctrl, control group.

At sacrifice, macrovesicular steatosis with ballooned hepatocytes, Mallory-Denk bodies, and foci of inflammatory cells, which are a recognized feature of NASH (Kleiner et al., 2005), were observed in the livers of rats in all groups, indicating that the histopathological characteristics that develop in this animal model reproduce those of NASH. However, these pathological effects were alleviated by the administration of 0.01% and 0.1% EGCG (Figure 1a and 1b). In particular the total NAFLD activity score (NAS) consisting of the steatosis, inflammation, and ballooning scores, was significantly lower in 0.1% EGCG-treated rats than in EGCG-untreated control rats (Figure 1c, *P* < 0.05). Similar results were obtained following measurement of intrahepatic lipid content: the levels of liver triglycerides in the DEN- and HFD-treated SD rats were significantly decreased when they received 0.01% and 0.1% EGCG instead of water (Figure 1d, *P* < 0.05). The serum alanine aminotransferase (ALT) levels were also significantly decreased with EGCG treatment at both concentrations relative to the levels in the water-treated group (Figure 1e, *P* < 0.05), indicating that EGCG protected against hepatic steatosis and subsequent hepatocyte injury induced by DEN and HFD.

### Effects of EGCG on liver fibrosis in rats

**Figure 2:**
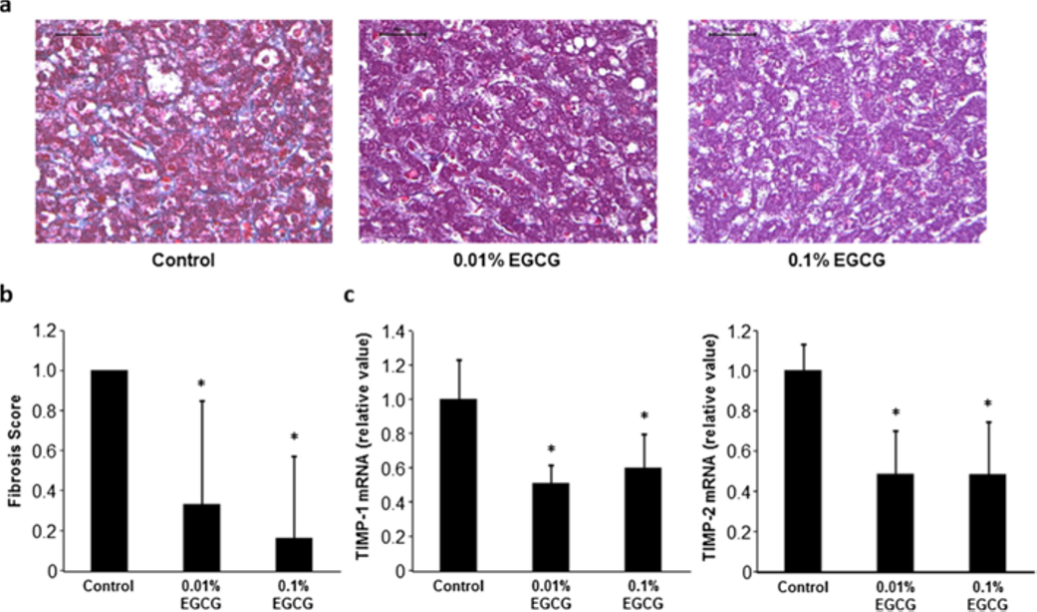
**Liver fibrosis and hepatic expression levels of TIMP-1 and TIMP-2 mRNA. (a)** Representative photomicrographs of Azan staining of liver tissues to show fibrosis. Bars, 50 μm. Note reduction of liver fibrosis (blue) by the treatment with EGCG. **(b)** Liver fibrosis evaluation was based on the NAS system. **(c)** After total RNA was isolated from the livers of the rats, the levels of TIMP-1 and TIMP-2 mRNA were measured using specific primers and quantitative real-time RT-PCR. The values are expressed as mean ± SD. * *P* < 0.05.

Examination of Azan-stained liver sections revealed that DEN- and HFD-treated SD rats developed perisinusoidal fibrosis, but that EGCG administration reduced liver fibrosis in the animals (Figure 2a). The liver fibrosis score was also significantly decreased by EGCG administration (Figure 2b). Furthermore, EGCG treatment significantly lowered (relative to control) the hepatic levels of TIMP-1 and TIMP-2 mRNA in the DEN- and HFD-treated SD rats (Figure 2c, *P* < 0.05).

### Effects of EGCG on the development of hepatic preneoplastic lesions in rats

**Figure 3:**
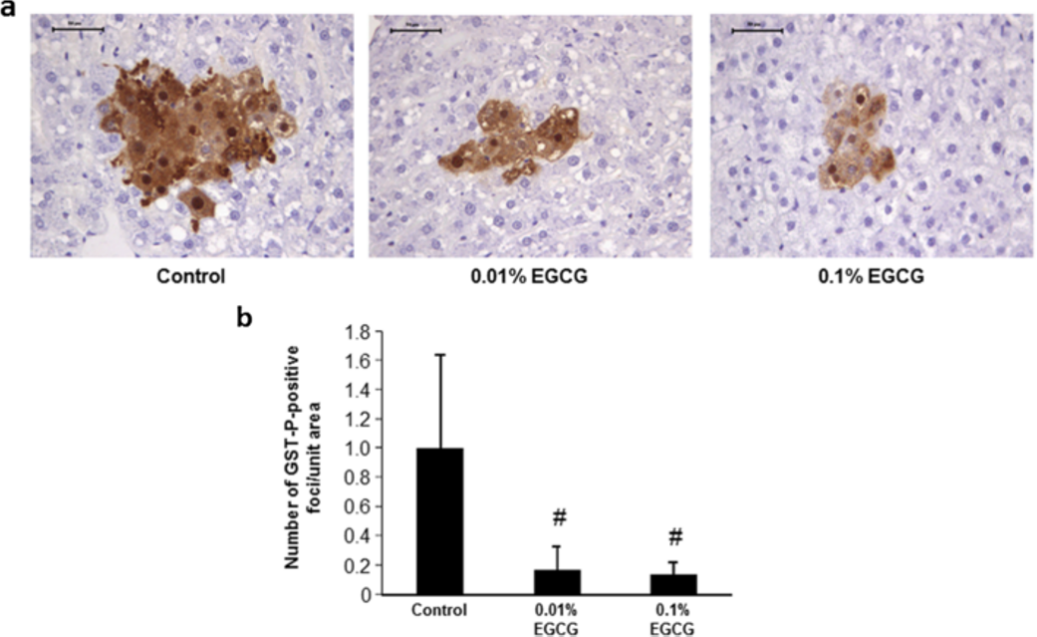
**The development of GST-P-positive foci in the livers. (a)** Representative photomicrographs of GST-P-positive foci. Note a large GST-P focus in a rat treated with DEN/HFD. Bars, 50 μm. **(b)** The average numbers of GST-P-positive foci formed in the livers of the rats in the 3 groups. The values are expressed as mean ± SD. # *P* < 0.01.

At the end of the experiment, GST-P-positive foci were detected in the livers of the rats, all of which had received DEN/HFD (Figure 3a). However, compared with rats in the control group that were not treated with EGCG, rats treated with EGCG showed a significant reduction in the number of GST-P-positive foci: 86% and 87% reduction relative to control following treatment with 0.01% and 0.1% EGCG, respectively (Figure 3b, *P* < 0.01).

### Effects of EGCG on oxidative stress in rats

**Figure 4:**
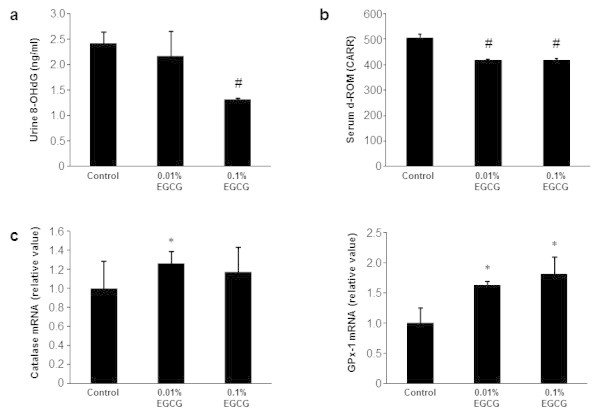
**Urinary 8-OHdG and serum d-ROM levels and on the hepatic catalase and GPx-1 mRNA levels. (a)** The urinary 8-OHdG levels were measured using ELISA. **(b)** Hydroperoxide levels in the serum were determined using the d-ROM test. **(c)** Levels of catalase and GPx-1 mRNA in the livers of the experimental rats were measured using specific primers and quantitative real-time RT-PCR. The values are expressed as mean ± SD. #*P* <0.01, *P <0.05.

Oxidative stress plays a critical role in the NAFLD-to-NASH progression and HCC development (Chiang et al., 2011; Cusi, 2012; Siegel and Zhu, 2009). Therefore, we examined the levels of oxidative stress and antioxidant biomarkers in the experimental rats. Rats administrated with 0.1% EGCG had significantly reduced levels of urinary 8-OHdG, a marker of DNA damage induced by oxidative stress, compared with EGCG-untreated control rats (Figure 4a, *P* < 0.01). The serum d-ROM level, which reflects serum hydroperoxide levels, was also decreased relative to control in rats treated with 0.01% and 0.1% EGCG (Figure 4b, *P* < 0.01). Moreover, the antioxidant enzyme (catalase and GPx-1) levels were significantly increased in the livers of rats that received EGCG treatment (Figure 4c, *P* < 0.05). These results indicate that drinking EGCG attenuated both systemic and hepatic oxidative stress in our rat model of NAFLD/NASH-related liver tumorigenesis.

### Effects of EGCG on hepatic expression of TNF-α, IL-6, and IL-1β mRNA in rats

**Figure 5:**
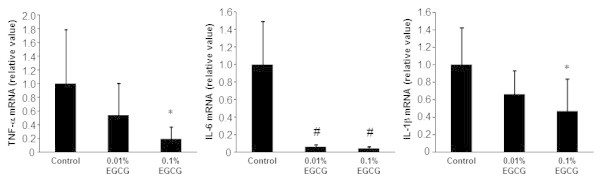
**Hepatic expression of TNF-α, IL-6, and IL-1β mRNA.** Total RNA was isolated from the livers of rats in the 3 groups and the expression levels of TNF-α, IL-6, and IL-1β mRNA were determined using specific primers and quantitative real-time RT-PCR. The values are expressed as mean ± SD. # *P* <0.01, *P <0.05.

Chronic inflammation is implicated in the progression of NASH and subsequent liver carcinogenesis (Chiang et al., 2011; Cusi, 2012; Siegel and Zhu, 2009; Park et al., 2010). Therefore, the mRNA expression levels of 3 inflammatory mediators, TNF-α, IL-6, and IL-1β, were measured in the livers of DEN- and HFD-treated SD rats. As shown in Figure 5, quantitative real-time RT-PCR analysis revealed that rats that received 0.1% EGCG exhibited significantly lower hepatic levels of TNF-α (*P* < 0.05), IL-6 (*P* < 0.01), and IL-1β (*P* < 0.05) mRNA than control rats that received only water, and the hepatic levels of IL-6 mRNA were also decreased by the administration of a low dose (0.01%) of EGCG (*P* < 0.01).

### Effects of EGCG on hepatocyte proliferation and hepatic expression of cyclin D1 mRNA in rats

**Figure 6:**
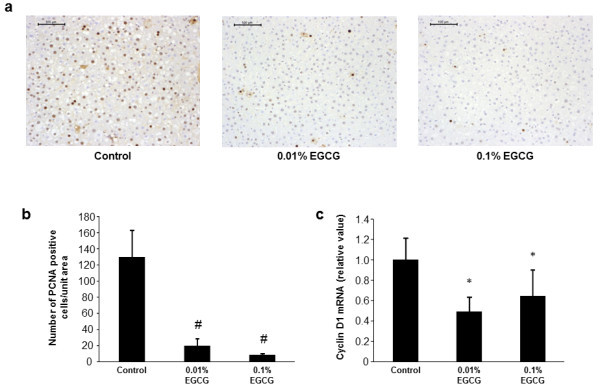
**Hepatocyte proliferation and hepatic expression of cyclin D1 mRNA. (a)** Immunohistochemical labeling for PCNA in the livers of the experimental rats. Bars, 100 μm. **(b)** The PCNA-labeling index in non-lesional hepatocytes was determined by counting the hepatocytes with PCNA-positive nuclei to calculate their percentage in the hepatocyte population. **(c)** The cyclin D1 mRNA levels in the liver of the experimental rats were determined using specific primers and quantitative real-time RT-PCR. The values are expressed as mean ± SD. # *P* <0.01, *P <0.05.

The PCNA-labeling index of non-lesional hepatocytes in DEN- and HFD-treated SD rats was determined based on the findings of immunohistochemical examination of sections (Figure 6a). The mean PCNA-labeling indices measured for rats administered 0.01% and 0.1% EGCG were significantly lower than that for EGCG-untreated control rats (Figure 6b, *P* < 0.01). Furthermore, the levels of cyclin D1 mRNA in liver were also markedly decreased in EGCG-treated rats relative to that in control rats (Figure 6c, *P* < 0.05), indicating that EGCG significantly inhibited hepatocyte proliferation in DEN- and HFD-treated SD rats.

## Discussion

An increase in the prevalence of NAFLD/NASH, which can progress to HCC, is a major healthcare problem worldwide (Chiang et al., 2011; Cusi, 2012; Siegel and Zhu, 2009). Therefore, developing an effective strategy for preventing NAFLD/NASH-related liver tumorigenesis is critical for improving the prognosis of patients with these diseases. The results of this study clearly indicate that EGCG, a GTC, effectively prevents the development of hepatic preneoplastic lesions, which manifest as GST-P-positive foci, in our rat model of NAFLD/NASH-related liver tumorigenesis. The rodent model used in this study, which was modified from a model described previously (Wang et al., 2009), reflects the pathological alterations implicated in NAFLD/NASH and NAFLD/NASH-related liver tumorigenesis, including the induction of oxidative stress and chronic inflammation (Chiang et al., 2011; Cusi, 2012; Siegel and Zhu, 2009; Park et al., 2010). Therefore, we consider the present model to be an appropriate and a useful animal model for analyzing the mechanisms of NAFLD/NASH-related liver tumorigenesis and for evaluating the efficacy of specific chemopreventive agents that can suppress such tumorigenesis.

Among the numerous pathophysiological conditions associated with NAFLD/NASH, oxidative stress is regarded as one of the key mechanisms for the development of HCC. In the progression of NAFLD to NASH, an increase in oxidative stress, which is defined as the overproduction of reactive oxygen species combined with inadequate anti-oxidative defense mechanisms, produces DNA damage and gene mutations associated with liver carcinogenesis (Chiang et al., 2011; Cusi, 2012; Siegel and Zhu, 2009; Park et al., 2010). Conversely, treatment with antioxidants such as vitamin E can reduce hepatic steatosis, lobular inflammation, and serum ALT levels, as shown by a clinical trial conducted in NASH patients (Sanyal et al., 2010). Administering pentoxifylline, which is known to decrease oxidative stress and inhibit TNF-α expression, also improved the histological features of NASH in a recent randomized placebo-controlled trial (Zein et al., 2011). In this study, treatment with EGCG lowered the levels of oxidative stress-associated markers such as urinary 8-OHdG and serum d-ROM, whereas elevated the mRNA levels of two antioxidant enzymes, catalase and GPx-1, in the liver of DEN- and HFD-treated SD rats. These findings suggest that EGCG suppressed NAFLD/NASH-related liver tumorigenesis at least partly by reducing systemic and hepatic oxidative stress. Our results are consistent with those of previous studies showing that GTCs can protect against both oxidative stress and the progression of NAFLD to NASH (Park et al., 2011; Ueno et al., 2009; Kuzu et al., 2008).

Besides oxidative stress, chronic inflammation is critically involved in NAFLD/NASH-related liver tumorigenesis (Chiang et al., 2011; Cusi 2012; Siegel and Zhu, 2009; Park et al., 2010). Among the proinflammatory cytokines related to the progression of NASH, TNF-α and IL-6 play a pivotal role in hepatocyte injury and inflammation, which increase HCC risk (Park et al., 2010). Therefore, targeting TNF-α and IL-6 might be an effective method to suppress NAFLD/NASH-related liver tumorigenesis. GTCs are widely recognized to exert cancer-preventive effects partly by inhibiting the expression of TNF-α and IL-6 (Shimizu et al. 2011b; Kochi et al., 2013; Shirakami et al. 2008), indicating that the suppression of inflammation is one of the key mechanisms by which GTCs prevent cancer development. In this study, we found that mRNAs encoding TNF-α, IL-6, and IL-1β were expressed at significantly lower levels in the livers of EGCG-treated rats compared to that in EGCG-untreated rats. Therefore, in agreement with previous reports (Shimizu et al. 2011b; Kochi et al., 2013; Shirakami et al. 2008), the results of this study suggest that EGCG consumption suppressed the development of GST-P-positive foci in DEN- and HFD-treated SD rats by attenuating chronic inflammation.

In addition to reducing oxidative stress and chronic inflammation, both of which are secondary manifestations of NASH, administering EGCG improved hepatic steatosis, a primary manifestation of NASH (Day and James, 1998), decreased serum ALT levels, ameliorated liver fibrosis, and inhibited excessive hepatocyte proliferation in this study. GTCs have been demonstrated to attenuate hepatic fat accumulation in several laboratory animal studies (Kuzu et al., 2008; Kochi et al., 2013; Shimizu et al. 2011b; Park et al., 2011; Ueno et al., 2009); these reports combined with the findings of this study are of interest because hepatic steatosis *per se* can induce hepatocyte proliferation and hepatic hyperplasia, both of which initiate the hepatic neoplastic process by increasing hepatocyte proliferative activity (Yang et al., 2001). Moreover, hepatic steatosis is critically related to liver fibrosis, which is a strong risk factor for the development of HCC (Powell et al., 2005). Therefore, suppression of hepatic steatosis and fibrosis by EGCG treatment might help to inhibit the progression of NAFLD/NASH-related liver tumorigenesis at an early stage.

Because GST-P-positive foci are generally accepted to be precursors or preneoplastic lesions of HCC in rats (Tsuda et al., 2003; Ando et al., 2007), the rodent model used in this study appears to be well suited for screening reagents that can prevent NAFLD/NASH-related liver tumorigenesis. However, the current study has one limitation that hepatocellular neoplasms, including HCC, did not develop within the experimental period. Because the duration of the experiment (7 weeks) might have been insufficient for the development of hepatic neoplasms, future studies should be conducted using longer-term experiments to confirm that HFD- and DEN-treated SD rats develop hepatocellular neoplasms.

Finally, we emphasize again that targeting metabolic abnormalities, especially oxidative stress and chronic inflammation, might be one of the most practical approaches for treating NAFLD/NASH and preventing NAFLD/NASH-related liver carcinogenesis (Shimizu et al. 2013,2012). We consider GTCs including EGCG to be potentially effective and key candidates for this purpose, because these agents can target metabolic abnormalities and thus prevent relevant tumorigenesis, as shown by the results of this study and those from previous reports (Shimizu et al. 2011b; Kochi et al., 2013; Shimizu et al. 2008b; Thielecke and Boschmann, 2009; Grove and Lambert, 2010). Recent clinical trials have also demonstrated that GTC supplementation potently prevents the development of both colorectal adenomas and prostate cancers without causing adverse effects (Shimizu et al. 2008a; Bettuzzi et al., 2006). These beneficial effects of GTCs reported in clinical trials strongly encourage the clinical usage of GTCs for treating NAFLD/NASH patients to prevent the metabolic abnormalities, such as steatosis, hyperlipidemia, and hyperinsulinemia, as well as liver carcinogenesis.

## Conclusions

In conclusion, administering EGCG effectively suppresses the early stage of hepatocarcinogenesis in our rat model of NAFLD/NASH by attenuating oxidative stress and chronic inflammation. Application of GTCs represents a potential new strategy for preventing the development of hepatic neoplasms in NAFLD/NASH patients.

## Methods

### Animals and chemicals

Male 7-week-old SD rats were obtained from Japan SLC, Inc. (Shizuoka, Japan) and humanely maintained at Gifu University Life Science Research Center in accordance with the Institutional Animal Care Guidelines. DEN was purchased from Sigma-Aldrich Co. LLC. (St. Louis, MO, USA). HFD-60 (HFD, 506.2 kcal/100 g) with 62.2% of the calories derived from fat was purchased from Oriental Yeast (Tokyo, Japan) (Table 2). EGCG was obtained from Mitsui Norin Co. Ltd. (Tokyo, Japan).

**Table 2:** **Composition and calories of the experimental diet HFD-60**

Ingredients	(g/kg diet)
Casein	256.0
Corn starch	160.0
Sucrose	55.0
Dextrose	60.0
Cellulose	66.1
Soybean oil	20.0
Lard	330.0
Vitamin mixture	35.0
Mineral mixture	10.0
Calcium carbonate	1.8
L-cysteine	3.6
Choline bitartrate	2.5
**Energy**	**(kcal/kg)**
	5062
	**(%)**
Protein	18.2
Fat	62.2
Carbohydrate	19.6

### Experimental procedure

The present study was approved by the Experimental Animal Research Committee of Gifu University. After 1-week-acclimatization with regular chow, all rats (n = 19) received a single intraperitoneal injection of DEN (30 mg/kg body weight) and were then randomly divided into 3 groups. Following DEN injection, the rats in Groups 2 and 3 (n = 6 in both groups) were provided tap water containing 0.01% or 0.1% EGCG, respectively, whereas the rats in Group 1 (n = 7) were provided tap water throughout the experiment, which lasted 7 weeks. All rats were fed a pelleted HFD throughout the experiment after DEN injection. At the end of the experiment, the 15-week-old rats were sacrificed by CO_2_ asphyxiation and the development of GST-P-positive foci was evaluated. The concentration of EGCG used (0.1%), which was established based on the findings of previous chemopreventive studies (Shimizu et al. 2011b; Kochi et al., 2013; Shirakami et al., 2008;2009), was, in terms of units per body weight, within the physiological range measured in humans after daily intake of GTCs (Wang et al., 1991). Previously, GST-P-positive foci were markedly induced by DEN injection in HFD fed rats, but not in rats fed a normal diet (Wang et al. 2009), therefore we did not use a control group that was fed normal chow diet after DEN injection in the present study.

### Histopathological examination and immunohistochemical analyses for GST-P and proliferating cell nuclear antigen (PCNA)

Maximum sagittal sections of 3 liver sublobes (central, lateral, and right-anterior) were used for histopathological examination. Formalin-fixed and paraffin-embedded livers were stained with hematoxylin & eosin (H&E) for conventional histopathology or with Azan stain to detect liver fibrosis. The histological features of the livers were evaluated using the NAFLD activity score (NAS) system (Kleiner et al., 2005), and the development of liver fibrosis was determined as described previously (Kleiner et al., 2005). Immunohistochemistry for GST-P (Ando et al., 2007) and PCNA (Iwasa et al., 2010) was performed using primary antibodies against GST-P (MBL Co. Ltd., Nagoya, Japan) and PCNA (Santa Cruz Biotechnology, Inc., Santa Cruz, CA, USA), respectively. The number of GST-P-positive foci, which was set as 3 or more positive cells (Kochi et al., 2013), was assessed per unit area (per cm^2^). In the PCNA-immunostained sections, cells with intensely stained nuclei were considered to be positive for PCNA, and the indices (% PCNA-positive) were determined by counting at least 500 hepatocytes in each section (total of 3000 hepatocytes per rat) (Iwasa et al., 2010).

### RNA extraction and quantitative real-time RT-PCR analysis

Total RNA was isolated from the livers of the rats by using RNeasy Mini kit (QIAGEN, Venlo, Netherlands) with on-column DNase I-digestion (Terakura et al., 2012). From 0.2 μg of total RNA, cDNAs were amplified using High-Capacity cDNA Reverse Transcription Kit (Applied Biosystems, Santa Clara, CA, USA) and an automated thermal cycler (Bio-Rad Laboratories, Hercules, CA, USA). Quantitative real-time reverse transcription-PCR (RT-PCR) analysis was performed using specific primers that amplify the TNF-α, IL-1β, IL-6, tissue inhibitor of metalloproteinases (TIMP)-1, TIMP-2, glutathione peroxidase (GPx)-1, catalase, cyclin D1, and glyceraldehyde-3-phosphate dehydrogenase (GAPDH) genes. The sequences of these primers, which were obtained using Primer-BLAST (http://www.ncbi.nlm.nih.gov/tools/primer-blast/), are shown in Table 3. Each sample was analyzed on a LightCycler Nano (Roche Diagnostics, Mannheim, Germany) with FastStart Essential DNA Green Master (Roche Diagnostics). GAPDH amplified in parallel served as the internal control.

**Table 3:** **Primer sequences**

Target gene	Direction	Primer sequences (5′-3′)
Catalase	Forward	GCGAATGGAGAGGCAGTGTAC
	Reverse	GAGTGACGTTGTCTTCATTAGCACTG
Cyclin D1	Forward	TTCGTGGCCTCTAAGATGAAGG
	Reverse	TGAGCTTGTTCACCAGAAGCAG
Gapdh	Forward	AGTGCCAGCCTCGTCTCATAG
	Reverse	CCTTGACTGTGCCGTTGAACT
Gpx-1	Forward	GCTCACCCGCTCTTTACCTT
	Reverse	GATGTCGATGGTGCGAAAGC
Il-1β	Forward	AGGCTTCCTTGTGCAAGTGT
	Reverse	TCCTGGGGAAGGCATTAGGA
Il-6	Forward	CACTTCACAAGTCGGAGGCT
	Reverse	AGCACACTAGGTTTGCCGAG
Timp-1	Forward	ACAGCTTTCTGCAACTCGGA
	Reverse	AGTTTGCAAGGGATGGCTGA
Timp-2	Forward	TGGGAACGTGCATTTTGCAG
	Reverse	AAACACTGGTTGGAGGGCAA
Tnf-α	Forward	CCAGACCCTCACACTCAGATCA
	Reverse	TCCGCTTGGTGGTTTGCTA

### Clinical chemistry

At sacrifice, the serum levels of ALT were measured using a standard clinical automatic analyzer (Type 7180; Hitachi, Tokyo, Japan).

### Hepatic lipid analysis

Total lipids were extracted from approximately 200 mg of liver tissue (frozen at sacrifice) for each rat, and the triglyceride levels were measured using the triglyceride E-test kit (Wako, Osaka, Japan) (Iwasa et al., 2010).

### Oxidative stress analysis

Urinary 8-hydroxy-2′-deoxyguanosine (8-OHdG) levels were determined using an ELISA kit (NIKKEN SEIL, Shizuoka, Japan). Serum levels of hydroperoxide, one of the markers for oxidative stress, were determined using the derivatives of reactive oxygen metabolites (d-ROM) test (FREE Carpe Diem; Diacron s.r.l., Grosseto, Italy) (Kochi et al., 2013).

### Statistical analysis

All data are expressed as mean ± SD, and one-way analysis of variance (ANOVA) was used for comparisons between groups. If ANOVA indicated significant differences, the Tukey-Kramer test for multiple comparisons was performed to compare the mean values among the groups. The differences were considered significant when the two-sided *P* value was less than 0.05. All analyses were conducted by the GraphPad InStat software, Version 3.05 (GraphPad Software; San Diego, CA, USA).

## Abbreviations

ALT: Alanine aminotransferase
ANOVA: Analysis of variance
Ctrl: Control
DEN: Diethylnitrosamine
d-ROM: Derivatives of reactive oxygen metabolites
EGCG: (-)-epigallocatechin-3-gallate
GAPDH: Glyceraldehyde-3-phosphate dehydrogenase
GPx: Glutathione peroxidase
GST-P: Glutathione *S*-transferase placental form
GTC: Green tea catechin
HCC: Hepatocellular carcinoma
HFD: High-fat diet
IL: Interleukin
NAFLD: Non-alcoholic fatty liver disease
NAS: NAFLD activity score
NASH: Non-alcoholic steatohepatitis
8-OHdG: 8-hydroxy-2′-deoxyguanosine
RT-PCR: Reverse transcription-PCR
SD: Sprague–Dawley
TG: Triglyceride
TIMP: Tissue inhibitor of metalloproteinase
TNF: Tumor necrosis factor.
